# Development and kinetic evaluation of vitamin C-loaded contact lenses prepared by a simple soaking technique

**DOI:** 10.3389/fmedt.2026.1759039

**Published:** 2026-02-26

**Authors:** Novan Rifky Lutfhyansyah, Haider Butt

**Affiliations:** 1 Department of Mechanical & Nuclear Engineering, Khalifa University of Science and Technology, Abu Dhabi, United Arab Emirates; 2 Advanced Digital & Additive Manufacturing (ADAM) Center, Khalifa University of Science and Technology, Abu Dhabi, United Arab Emirates; 3 Food Security and Technology Center (FSTC), Khalifa University of Science and Technology, Abu Dhabi, United Arab Emirates

**Keywords:** contact lenses, drug delivery, mathematical models, release kinetics, vitamin C

## Abstract

Conventional ocular drug delivery systems are often limited by low bioavailability and short residence times at the ocular surface, motivating the development of alternative delivery platforms. In this study, vitamin C-loaded contact lenses were prepared using a simple soaking technique and systematically evaluated. Two commercial lenses, Senofilcon A and Hilafilcon B, were immersed in vitamin C solutions, and their loading efficiency, release kinetics, stability, water content, and oxygen permeability were investigated. Vitamin C release was quantified using UV-Vis spectrometry and analyzed using zero-order, first-order, Higuchi, and Korsmeyer–Peppas models. Hilafilcon B exhibited higher vitamin C loading and cumulative release (∼14 µg/mL) than Senofilcon A (∼10 µg/mL), consistent with its higher hydrophilicity and equilibrium water content (∼56% compared to ∼29%). Kinetic analysis indicated that the vitamin C release from both lenses was best described by the Korsmeyer–Peppas model (Hilafilcon B, *n* = 0.610; Senofilcon A, *n* = 0.783), suggesting anomalous transport behavior. Vitamin C stability was strongly influenced by storage conditions, with refrigerated storage improving stability, while UV exposure accelerated degradation. Overall, these findings demonstrate that vitamin C incorporation via soaking provides a straightforward approach for developing antioxidant-loaded contact lenses with potential relevance for ocular drug delivery.

## 1 Introduction

Conventional topical drug delivery methods for ophthalmic applications, such as eye drops, face significant challenges due to low bioavailability and poor ocular drug penetration. These limitations are largely due to the eye's natural defense mechanisms, including tear turnover, blinking, and various anatomical barriers that restrict drug absorption (1–3). As a result, only a small fraction, typically between 1%–5%, of the administered dose actually reaches intraocular tissues.

To compensate for these limitations, higher drug concentrations and frequent administration are often required. This not only increases the risk of local and systemic side effects but also negatively affects patient compliance, especially in chronic conditions like glaucoma or dry eye syndrome (3–5). The inefficiency of traditional eye drop formulations has prompted the development of alternative drug delivery systems aimed at enhancing therapeutic efficacy and patient convenience.

One promising alternative is the use of drug-eluting contact lenses. These devices can deliver drugs directly and continuously to the ocular surface, significantly enhancing bioavailability while minimizing systemic exposure (6, 7). Furthermore, drug-eluting contact lenses offer considerable design flexibility, allowing customization of lens materials, drug type, and loading strategies to address specific ocular conditions and patient-specific therapeutic requirements, similar to other polymer-based drug delivery systems (6, 8, 9). Overall, compared to eye drops, contact lenses provide a larger surface area for drug absorption and can extend drug residence time, potentially reducing the frequency of administration and thereby improving therapeutic efficacy.

Vitamin C (ascorbic acid) is a vital compound for maintaining ocular health due to its potent antioxidant properties and exceptionally high concentration in the eye. The vitamin C level in the aqueous humor ranges between 1 and 2 mM, approximately 40 times higher than in the blood, accounting for about 75% of the total antioxidant potential in this region (10). This elevated concentration highlights its crucial role in neutralizing reactive oxygen species (ROS) and safeguarding ocular structures, including the cornea, lens, and retina, from oxidative damage. Reduced levels of vitamin C have been detected in individuals suffering from ocular disorders such as cataracts, Lowe's syndrome, exfoliation syndrome, and glaucoma (11). Studies have shown that vitamin C helps prevent oxidative stress-related ocular diseases, including cataracts, glaucoma, and age-related macular degeneration (12–14). For example, vitamin C has been reported to protect the lens and trabecular meshwork by consuming oxygen in the vitreous, thereby reducing oxidative degradation (10). Clinically, its therapeutic potential has been observed in animal corneal health, where topical vitamin C promoted wound healing and prevented endothelial cell loss after cataract surgery (15, 16). Moreover, its biological role extends to stimulating collagen type I synthesis, supporting epithelial regeneration, and enhancing tissue repair capacity (17). Altogether, these findings emphasize that vitamin C is not merely an antioxidant but a multifunctional molecule indispensable for preserving ocular integrity, preventing oxidative injury, and maintaining long-term visual health.

Despite its therapeutic importance, effective ocular delivery of vitamin C remains challenging due to limitations of both systemic and topical administration. Systemic delivery must overcome numerous static and dynamic ocular barriers, including the blood-aqueous and blood-retinal barriers, which restrict the passage of circulating compounds into ocular tissues (18, 19). Conventional eye drops also demonstrate low bioavailability, which can limit therapeutic effectiveness. Additionally, vitamin C solutions are susceptible to oxidative degradation, which further reduces the concentration of active vitamin C (20). Accordingly, drug-eluting contact lenses offer a potential strategy to enhance local delivery by extending residence time on the ocular surface and minimizing early oxidation of vitamin C.

The drug release behavior from polymeric systems is typically analyzed using mathematical models, such as zero-order, first-order, Higuchi, and Korsmeyer-Peppas models, to determine the underlying release mechanisms. Zero-order kinetics describe a constant drug release rate that is independent of drug concentration, which is ideal for maintaining stable therapeutic levels (21). First-order kinetics indicate that the release rate is proportional to the remaining drug content within the system (21). The Higuchi model explains drug release as a diffusion-controlled process based on Fick's law, commonly applicable to systems where the drug diffuses through a porous or swollen matrix (21). The Korsmeyer-Peppas model provides further mechanistic insight by distinguishing between Fickian diffusion, anomalous transport (a combination of diffusion and polymer relaxation), and case II transport (polymer relaxation or swelling-controlled release) through the release exponent (21). Collectively, these models help elucidate how the properties of the polymer matrix and lens material influence drug release profiles, which is critical for the rational design and optimization of drug-eluting contact lenses (22–24). Applying these models enables a deeper understanding of vitamin C release performance and supports the development of improved formulations in future studies.

Although research on drug-eluting contact lenses has advanced considerably in recent years, to the best of our knowledge, no studies have reported the incorporation of vitamin C into contact lenses for sustained ocular delivery. Given vitamin C's established role in ocular protection and tissue repair, this represents an unexplored yet promising direction within the broader field of therapeutic contact lenses. Therefore, this study aims to develop vitamin C-loaded contact lenses utilizing a straightforward soaking technique. We examine the incorporation efficiency and drug release kinetic profile of vitamin C from the commercial lenses. Additionally, we assess the stability of vitamin C under various conditions to optimize the loading method into contact lenses. Furthermore, we measure the water content to evaluate the impact of vitamin C addition on the lenses' water retention capacity and oxygen permeability. The present study aims to establish a foundational approach for the utilization of vitamin C in future therapeutic applications in ophthalmology.

## 2 Materials and methods

**Figure 1:**
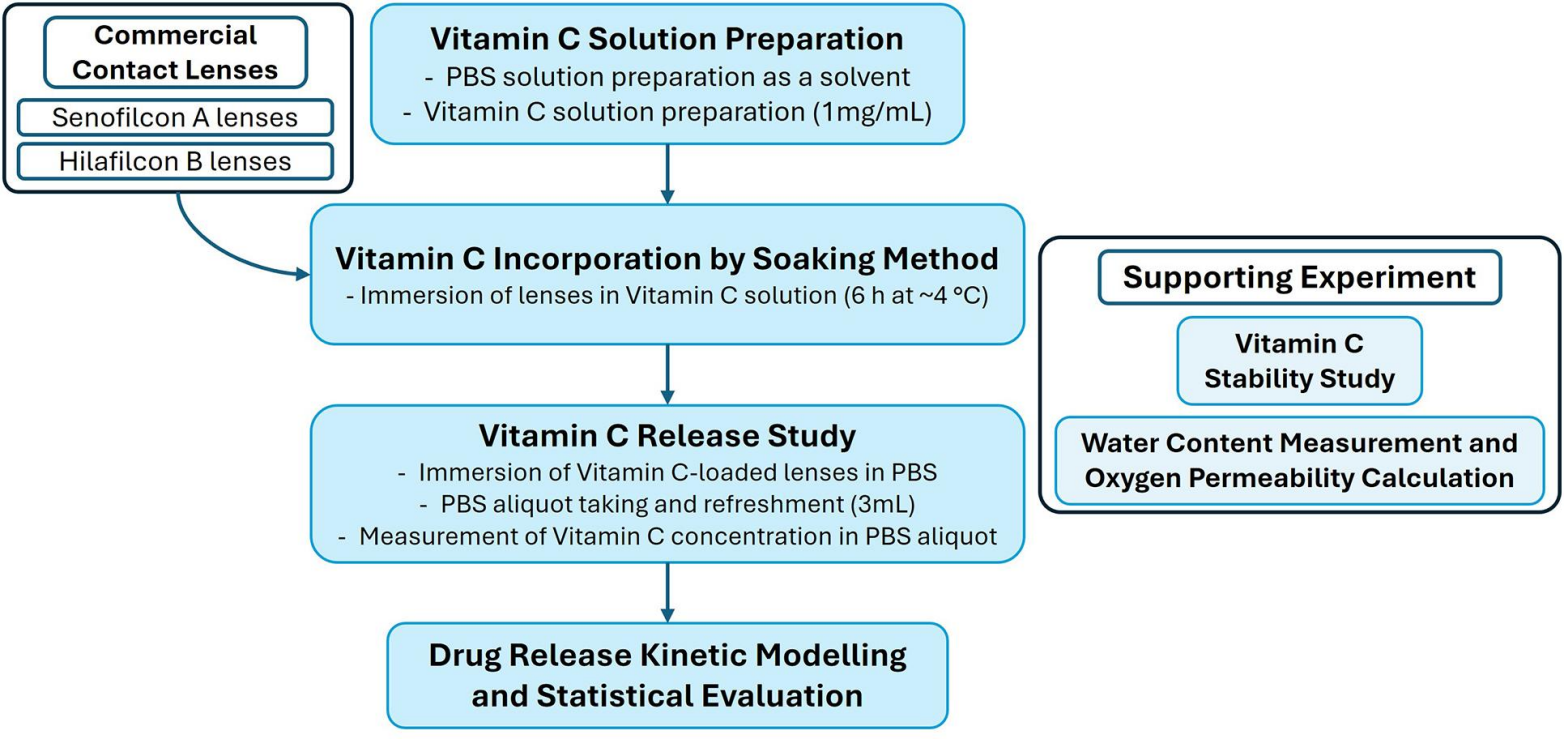
Overview of the experimental workflow, including vitamin C solution preparation, incorporation into commercial contact lenses, release study, and supporting analyses for stability, water content, and oxygen permeability.

Figure 1 presents an overview of the experimental workflow conducted in this study, including the preparation of the drug solution, the incorporation of the drug into commercial contact lenses, the drug release study, and supporting analyses for stability, water content, and oxygen permeability. The details of each component are provided in the following sections.

### 2.1 Materials

In this study, two types of contact lenses were utilized. There are Senofilcon A from Acuvue Oasys with Hydraluxe (Dia 14.3 mm, BC 8.5 mm, *P* −0.75) and Hilafilcon B from Bausch & Lomb Soflens (Dia 14.2 mm, BC 8.6 mm, *P* −1.00). L-Ascorbic Acid or vitamin C was obtained from Sigma-Aldrich. Moreover, phosphate-buffered saline (PBS) in the form of tablets (pH 7.3 ± 0.2 at 25°C) was obtained from Oxoid. All chemicals were used as received without any further treatment.

### 2.2 Vitamin C loading into contact lenses

**Figure 2:**
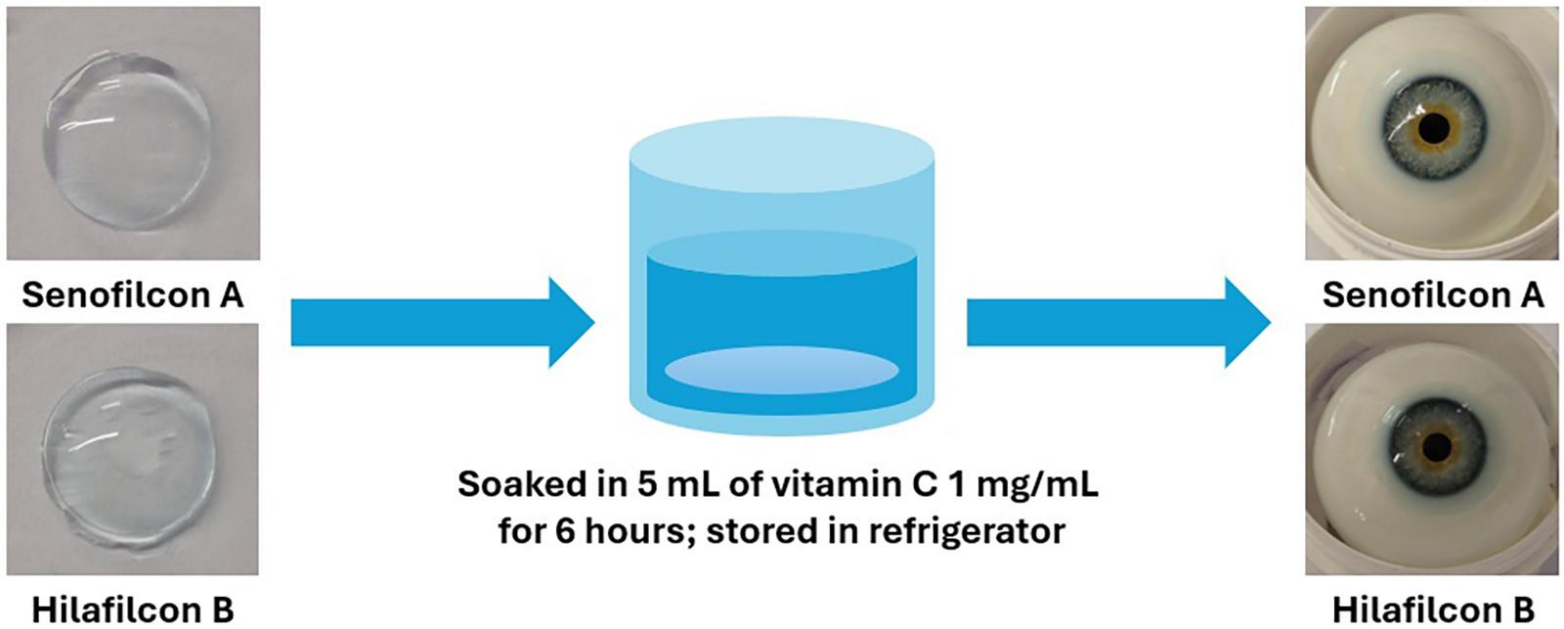
Contact lenses before and after vitamin C loading.

To make the vitamin C solution, the PBS solution was prepared as the solvent. A single PBS tablet was dissolved in 100 mL of deionized water, followed by heating at 115°C for 10 min. The vitamin C solution was formulated by dissolving vitamin C powder in the PBS solution. To prepare the vitamin C-loaded contact lenses, the lenses were immersed in a 1,000 µg/mL (1 mg/mL) vitamin C solution for 6 h and subsequently stored in a refrigerator (∼4°C). The soaking duration and temperature were selected to allow the lenses to reach equilibrium with the vitamin C solution while minimizing premature oxidation. Figure 2 shows the lenses before and after the incorporation of vitamin C.

### 2.3 Vitamin C release study

The vitamin C-loaded contact lenses were immersed in a small jar containing 5 mL of PBS solution. To simulate tear fluid drainage and renewal, a portion of the PBS was periodically withdrawn and replaced with fresh PBS every 30 min. Specifically, 3 mL of the solution was removed and replenished with an equal volume of fresh PBS at each interval. The collected aliquots were analyzed using a UV-Vis spectrometer (Ocean Insight Miniature Spectrometer) to monitor the release of vitamin C from the contact lenses. The release study was performed at 30 min intervals for a total duration of 3 h. The concentration of vitamin C in each aliquot was determined based on a predetermined calibration curve of vitamin C solutions. All release experiments were performed in triplicate (*n* = 3 for each commercial lens type).

### 2.4 Kinetic release model of vitamin C release

Different mathematical models were applied to evaluate the kinetic release profiles of the samples over the studied time duration. These included zero-order and first-order release models, the Higuchi release model, and the Korsmeyer-Peppas model. The graphs are plotted, and the coefficient of determination (R^2^) of each model is determined according to the equation of each release model, as shown in Table 1 (25–28). Additionally, the residual sum of squares (RSS), Akaike information criterion (AIC), and model selection criterion (MSC) are calculated to evaluate the validity of each model fitting.

**Table 1:** Various drug release kinetic model equations.

Parameter	Zero order	First order	Higuchi model	Korsmeyer-Peppas model
Equation	$C_{t}=\;C_{0}+\;K_{0}t$	$\log\;C_{t}=\log\;C_{0}-\left(\frac{K_{1}}{2.303}\right)t$	$\frac{M_{t}}{M_{\infty }}=\;K_{h}t^{1/2}$	$\log\;\left(\frac{M_{t}}{M_{\infty }}\right)=\log\;K_{kp}-n\log\;t$
Graph	Cumulative drug release vs. time	log cumulative % drug remaining vs. time	Cumulative % drug release vs. $\sqrt{t}$	log cumulative % drug release vs. log t

### 2.5 Vitamin C stability study

To investigate the stability of vitamin C under different storage conditions, two solutions of 10 µg/mL vitamin C were prepared and stored either in a closed drawer at room temperature (∼22 °C) and inside a refrigerator (∼4°C). The UV spectra were recorded before and after a 24 h storage period. Additionally, the photostability of vitamin C was examined by exposing a 10 µg/mL solution to UV light (*λ* = 365 nm) using a UV chamber (Analytik Jena, Germany). The spectra were collected at 60 min intervals over the exposure period. All UV stability tests were performed at room temperature.

### 2.6 Water content measurement

The water content study is conducted to see the effect of vitamin C on the water uptake capacity of the contact lenses. The lenses were dried using an oven at 60°C for 60 min, and then the dry weight was measured. Then, the lenses are soaked in DI water, PBS solution, and vitamin C solution for a particular time, and the weight is measured as the wet weight. Each measurement was performed in triplicate for both lens types (*n* = 3). The water content of the contact lenses can be calculated by the following equation:

$$\mathrm{Water}\;\mathrm{content}\;(\text{\%})=\frac{\mathrm{Wet}\;\mathrm{weight}-\mathrm{Dry}\;\mathrm{weight}}{\mathrm{Wet}\;\mathrm{weight}}\;\times 100\text{\%}$$

Additionally, the oxygen permeability (Dk) of each lens was estimated using the Morgan and Efron empirical relationship, expressed as Dk = 1.67e^0.00397EWC^, where EWC is the equilibrium water content of the material (9, 29).

### 2.7 Statistical evaluation

All quantitative measurements were performed in triplicate (*n* = 3). Statistical comparisons between Senofilcon A and Hilafilcon B were conducted using unpaired two-sample t-tests, with *p* < 0.05 considered statistically significant. Statistical analyses were performed using Microsoft Excel.

## 3 Results and discussion

**Figure 3:**
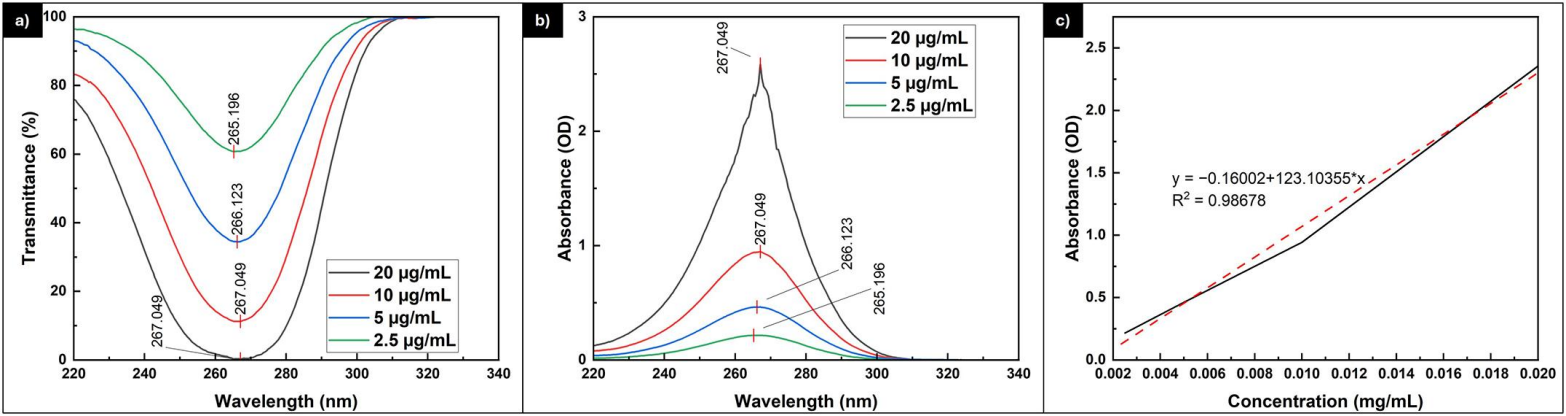
The **(a)** transmittance and **(b)** absorbance of vitamin C solution with various concentrations to construct the **(c)** standard curve of vitamin C solution.

The detection of the vitamin C molecule can be accomplished using a UV spectrometer, which reveals a characteristic peak at around 265 nm. Figure 3 displays the transmittance and absorbance of various concentrations of vitamin C solution. Additionally, a standard curve was constructed from the absorption data, allowing for the determination of unknown vitamin C concentrations. The figure clearly shows that the peaks indicating vitamin C fall within the range of 265.196 nm to 267.049 nm, similar to previously reported values in the literature (30–32). As the concentration increases, the peak wavelength also rises. While variations in peak wavelength are common, this observation might imply that a higher concentration of vitamin C requires slightly less energy to induce molecular vibrations.

**Figure 4:**
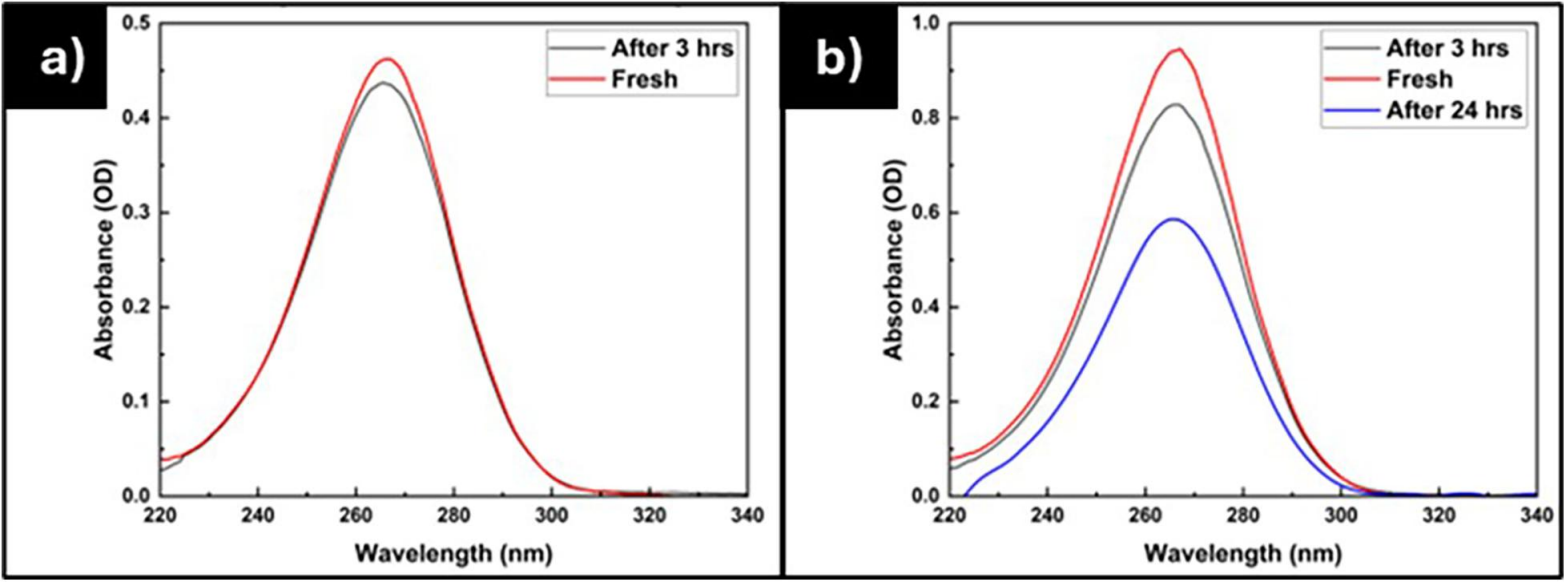
The absorbance of the vitamin C solution with **(a)** 5 µg/mL stored in a fridge and **(b)** 10 µg/mL stored at room temperature.

**Figure 5:**
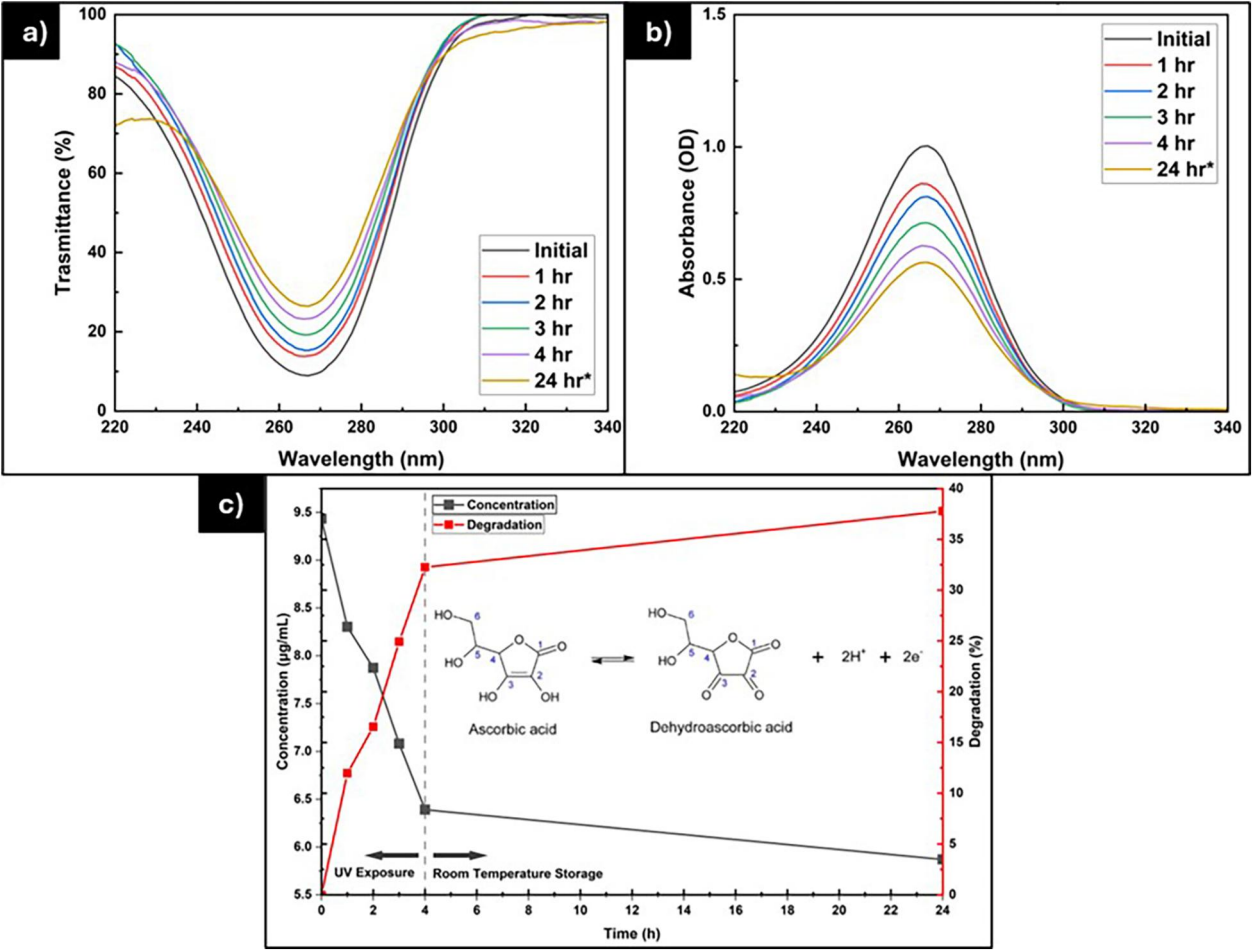
The **(a)** transmittance and **(b)** absorbance of vitamin C solution after UV exposure. The **(c)** concentration and degradation percentage of vitamin C solution during the stability test.

Vitamin C is prone to degradation, especially when exposed to light and oxygen. Research has demonstrated that storage conditions greatly influence the stability of vitamin C (20, 33, 34). Consequently, we examined how different storage conditions affect the stability of vitamin C solutions, as illustrated in Figure 4. Furthermore, we also explored the stability of vitamin C when exposed to UV light, as depicted in Figure 5 and Table 2.

**Table 2:** The concentration of vitamin C after UV exposure.

Time	Concentration (μg/mL)	Degradation (%)
Initial	9.434	0
1 h	8.304	11.98
2 h	7.874	16.54
3 h	7.082	24.93
4 h	6.391	32.26
24 h*	5.871	37.77

* = measured at 24 h after solution preparation; UV exposure was stopped after 4 h.

The effect of storage temperature on the degradation of vitamin C is illustrated in Figure 4. Figure 4a presents the absorbance of a 5 µg/mL vitamin C solution stored in a refrigerator, while Figure 4b displays the absorbance of a 10 µg/mL vitamin C solution stored at ambient temperature. After a duration of 3 h, the 5 µg/mL vitamin C solution stored in the refrigerator exhibits a lesser degree of degradation, with the concentration decreasing by approximately 2.99% from the initial concentration. In contrast, the 10 µg/mL vitamin C solution stored at room temperature shows a reduction of up to 19.77% from its initial concentration. Furthermore, after 24 h, the 10 µg/mL vitamin C solution degrades by up to 40.03%. These findings suggest that the optimal condition for employing the soaking method to load vitamin C into lenses is best achieved when conducted in a refrigerated environment.

Figures 5a,b show the absorbance and transmittance spectra of the vitamin C solution before and after UV exposure, while Figure 5c and Table 2 summarize the corresponding changes in concentration and degradation percentage. The results clearly indicate that UV irradiation accelerated the degradation of vitamin C. After 4 h of exposure to 365 nm UV light, the vitamin C concentration decreased by 32.26%, whereas during 20 h of storage at room temperature, only about 5% degradation occurred, leaving 37.77% of the initial concentration. This demonstrates that UV radiation substantially enhanced the rate of vitamin C loss. The degradation mechanism is likely governed by indirect oxidative degradation, in which UV exposure generates reactive oxygen species (ROS) such as singlet oxygen and hydroxyl radicals from dissolved oxygen or residual impurities in the medium. These ROS subsequently oxidize ascorbic acid to dehydroascorbic acid and other breakdown products. Hence, the degradation is not primarily caused by direct photolysis, since ascorbic acid strongly absorbs at ∼265 nm, whereas the UV lamp used in this study emits at 365 nm. Aguilar et al. (2019) similarly reported that UV-Vis irradiation did not significantly accelerate ascorbic acid degradation in aqueous solutions due to its low absorption within the lamp's emission range (35). Nevertheless, other studies have shown that UV exposure generally promotes ascorbic acid degradation through photo-oxidative mechanisms, aligning with the present findings (36–39).

**Figure 6:**
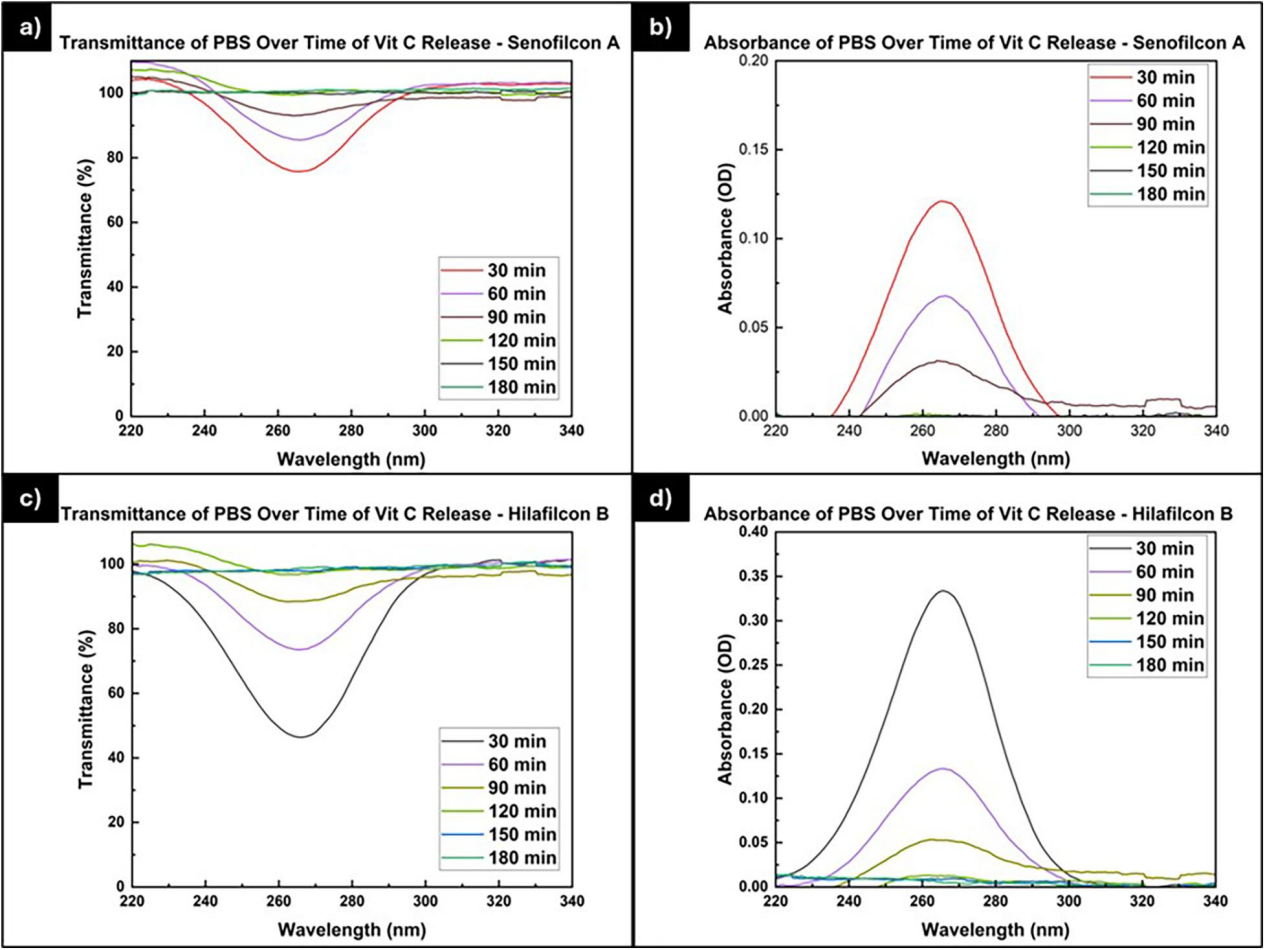
The **(a)** transmittance and **(b)** absorbance of PBS for the vitamin C release study for Senofilcon A; the **(c)** transmittance and **(d)** absorbance of PBS for the vitamin C release study for Hilafilcon B.

The transmittance and absorbance data for the vitamin C release study are presented in Figure 6. The graphs clearly demonstrate that the peaks indicating vitamin C transmission in Hilafilcon B are lower than those in Senofilcon A. This suggests that Hilafilcon B has a higher capacity to absorb vitamin C compared to Senofilcon A. However, after 90 min, no significant peaks are observed for either Senofilcon A or Hilafilcon B. Therefore, it implies that the vitamin C release rate of Hilafilcon B is greater than that of Senofilcon A.

**Figure 7:**
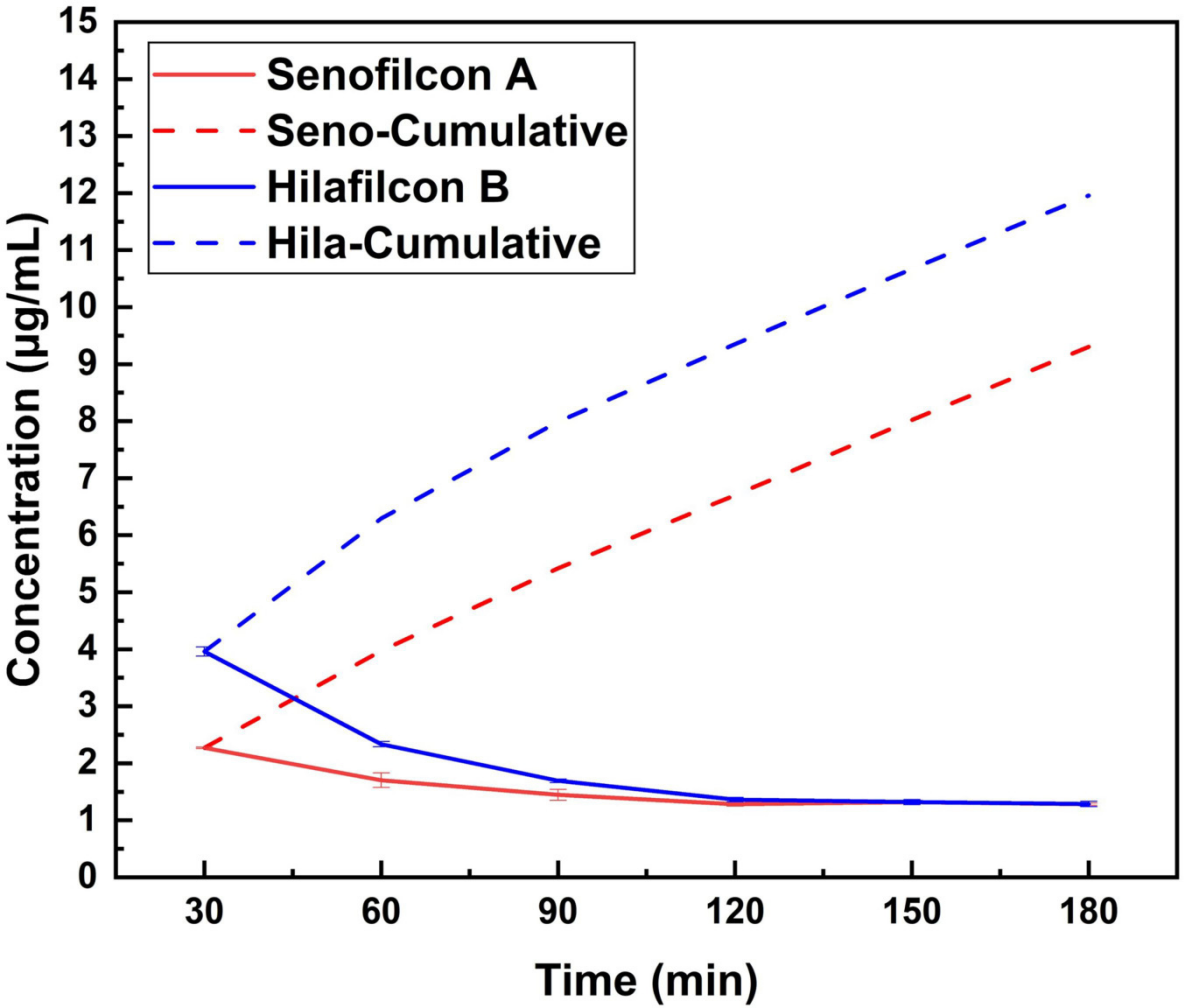
Vitamin C release from Senofilcon A and Hilafilcon B lenses.

Based on the absorbance measurements at different time intervals, the concentration of vitamin C released from both Senofilcon A and Hilafilcon B lenses was determined, as shown in Figure 7 and summarized in Table 3. Both materials exhibited an initial burst release followed by a gradual decline in the release rate over time. However, statistical comparison using an unpaired t-test showed that Hilafilcon B released significantly more vitamin C than Senofilcon A at the early (30 min), mid (120 min), and final (180 min) timepoints (*p* < 0.05 for all comparisons). After 180 min, Hilafilcon B reached a cumulative concentration of approximately 14 µg/mL, whereas Senofilcon A reached about 10 µg/mL. This indicates that Hilafilcon B released vitamin C more rapidly and in greater quantities in the initial steps than Senofilcon A, likely due to its higher water content and greater hydrophilicity, which facilitate faster molecular diffusion through the hydrogel matrix. After 90 min, the release from both lenses began to plateau, suggesting that the diffusion process approached equilibrium as the concentration gradient diminished.

**Table 3:** Summary of vitamin C concentration released from Senofilcon A and Hilafilcon B.

Time (min)	Senofilcon A		Hilafilcon B	
	Concentration (μg/mL)	Cumulative concentration (μg/mL)	Concentration (μg/mL)	Cumulative concentration (μg/mL)
30	2.270 ± 0.012	2.270	3.960 ± 0.082	3.960
60	1.703 ± 0.126	3.973	2.336 ± 0.046	6.296
90	1.446 ± 0.096	5.419	1.693 ± 0.032	7.989
120	1.282 ± 0.032	6.701	1.362 ± 0.039	9.351
150	1.322 ± 0.043	8.022	1.320 ± 0.044	10.671
180	1.283 ± 0.021	9.305	1.288 ± 0.047	11.959

**Figure 8:**
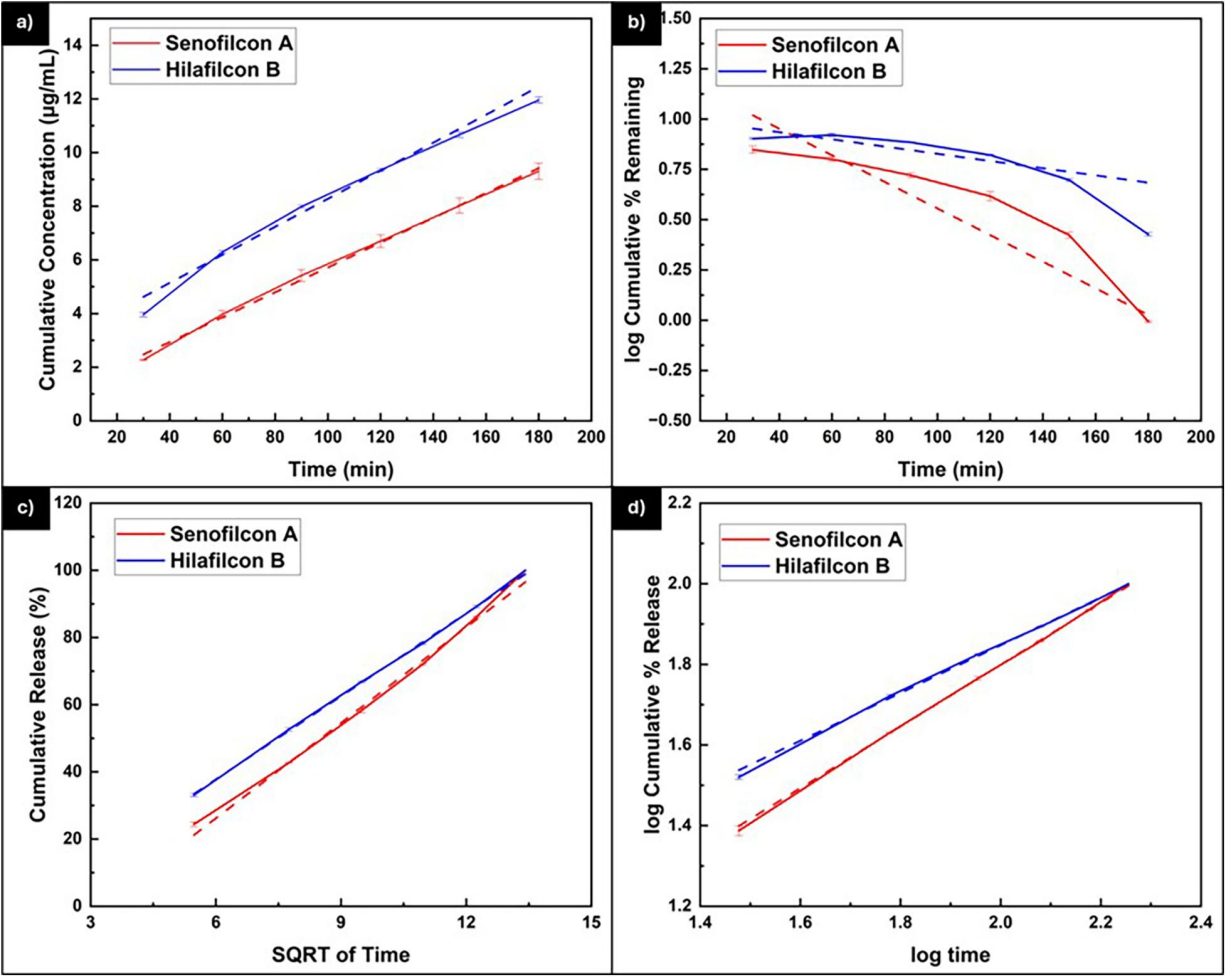
Kinetic model of **(a)** zero-order, **(b)** first-order, **(c)** Higuchi model, and **(d)** Korsmeyer-peppas model of vitamin C release from Senofilcon A and Hilafilcon B lenses.

Figure 8 illustrates the fitting of the experimental data to different kinetic models describing the release of vitamin C from Senofilcon A and Hilafilcon B lenses, with the corresponding statistical parameters summarized in Table 4. Both lenses demonstrated strong correlations (R² ≥ 0.95) for all models except the first-order model, indicating that concentration-dependent kinetics do not adequately represent the release behavior of vitamin C in these systems. However, such high R² values should be interpreted with caution, as strong correlations can sometimes reflect model overfitting rather than true mechanistic accuracy. Moreover, the Higuchi and Korsmeyer-Peppas models exhibited the best agreement with the experimental data, suggesting that the release process is primarily governed by diffusion through the hydrogel matrix. According to the Korsmeyer-Peppas model, the release exponent (n) for both lenses lies within the range of 0.5 < *n* < 1, indicating that the mechanism follows anomalous transport, where both diffusion of vitamin C and polymer relaxation contribute to the overall release (21, 40–42). The lower *n* value for Hilafilcon B (*n* = 0.610) compared to Senofilcon A (*n* = 0.783) implies that the release from Hilafilcon B aligns more closely with Fickian diffusion, whereas Senofilcon A implies a more balanced contribution from diffusion and polymer matrix swelling or relaxation. Overall, based on the trends observed in the model fitting, these results suggest that the release of vitamin C from both materials is primarily diffusion-controlled, with the relative influence of relaxation or swelling effects varying between lens types.

**Table 4:** Summary of the release kinetic statistical evaluation of Senofilcon A and Hilafilcon B.

Sample	Parameter	Zero-order	First-order	Higuchi	Korsmeyer-Peppas
Senofilcon A	Linear regression equation	y = 0.0463x + 1.0875	y = −0.0052x + 2.1485	y = 9.4827x − 30.7273	y = 0.7826x + 0.2341
	K	0.046	0.005	9.483	1.714
	R^2^	0.996	0.851	0.998	1.000
	RSS	19.590	8.977	2,113.556	0.114
	AIC	11.100	6.417	39.186	−19.782
	MSC	−0.120	−3.539	−0.052	0.133
	*n*	NA	NA	NA	0.783
Hilafilcon B	Linear regression equation	y = 0.0519x + 2.9228	y = −0.003x + 2.0094	y = 8.2376x − 11.6376	y = 0.6104x + 0.6259
	K	0.052	0.003	8.238	4.226
	R^2^	0.982	0.775	0.9995	0.999
	RSS	25.101	6.372	1,486.068	0.070
	AIC	12.587	4.361	37.073	−22.747
	MSC	−0.128	−4.238	0.039	0.132
	*n*	NA	NA	NA	0.610

K, rate constant; R^2^, coefficient of determination; RSS, residual sum square; AIC, Akaike information criterion; MSC, model selection criterion; *n*, release exponential coefficient.

The statistical evaluation further supports the analysis of vitamin C release behavior from both lenses (40). The rate constant (K) of Senofilcon A was found to be slightly higher than that of Hilafilcon B across all kinetic models, indicating a somewhat faster initial release rate from Senofilcon A. However, Hilafilcon B ultimately achieved a higher cumulative vitamin C concentration, which can be attributed to its greater loading capacity and higher water content that facilitate solute retention and diffusion over time. To minimize the risk of overfitting, AIC values were considered in conjunction with R². Based on the R² and Akaike Information Criterion (AIC) values, both lenses exhibited lower AIC and higher R² values for the Korsmeyer-Peppas model compared to the Higuchi model, signifying a superior fit and greater predictive reliability. This finding further suggests that the release of vitamin C from both Senofilcon A and Hilafilcon B is governed by a combination of Fickian diffusion and polymer matrix swelling or relaxation, consistent with an anomalous transport mechanism rather than purely diffusion-controlled behavior.

**Figure 9:**
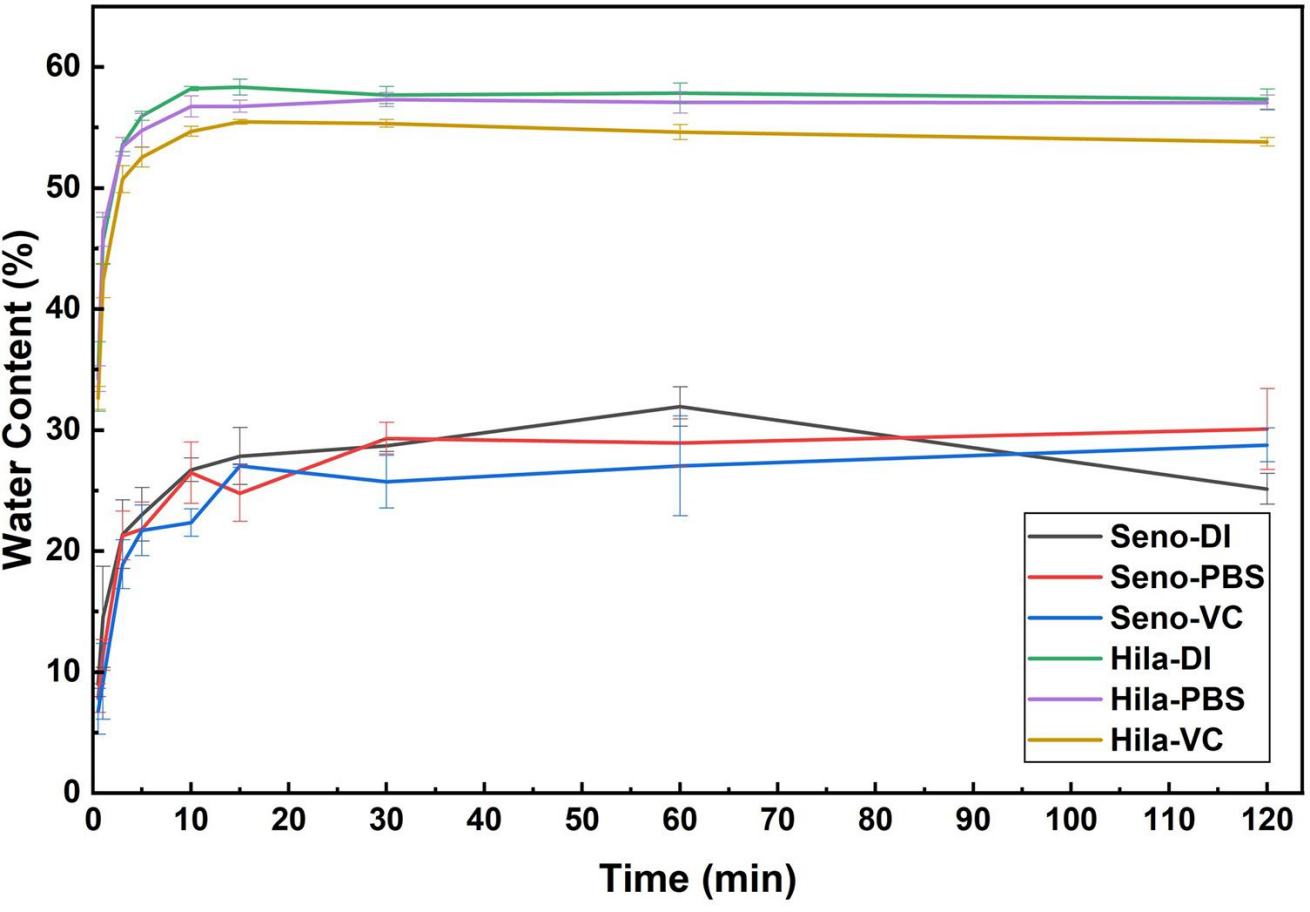
Water content of Senofilcon A and Hilafilcon B after soaking in DI water, PBS solution, and vitamin C solution.

To evaluate the effect of vitamin C incorporation on the hydration behavior and oxygen permeability of the lenses, water content analysis was conducted, as illustrated in Figure 9 and summarized in Table 5. Hilafilcon B exhibited a statistically significant and substantially higher water content than Senofilcon A across all media (*p* < 0.05, unpaired t-test). The equilibrium water content of Hilafilcon B reached approximately 55%–57% and stabilized within the first 30 min across all media, whereas Senofilcon A exhibited a lower equilibrium range of 27%–32% with slightly slower hydration, particularly in PBS and vitamin C solutions. The incorporation of vitamin C resulted in a small decrease in water content compared to DI water and PBS for both lens types. However, statistical analysis indicated that this difference was significant only for Hilafilcon B, while the change observed for Senofilcon A was not statistically significant. Overall, the magnitude of the water content reduction was modest, suggesting that vitamin C incorporation does not substantially alter the hydration behavior of the lenses.

**Table 5:** Summary of water content and oxygen permeability value of Senofilcon A and Hilafilcon B in different solutions.

Sample	Water content (%)	Oxygen permeability, Dk (barrer)
Seno-DI	31.939 ± 1.636	5.935
Seno-PBS	28.931 ± 1.964	5.267
Seno-VC	27.039 ± 4.133	4.886
Hila-DI	57.373 ± 0.325	16.289
Hila-PBS	56.581 ± 0.307	15.785
Hila-VC	54.604 ± 0.626	14.594

The observed changes in water content were accompanied by a corresponding decrease in estimated oxygen permeability (Dk). Hilafilcon B displayed higher Dk values (14.6–16.3 barrer) than Senofilcon A (4.9–5.9 barrer), consistent with oxygen diffusion occurring primarily through hydrated channels in the hydrogel and through the silicone phase in the silicone hydrogel. It should be noted that the oxygen-permeability values reported here are estimated using an empirical water-content-based equation and, therefore, may not fully represent the true oxygen-transport properties of the lenses (9). Nonetheless, the greater reductions in both water content and estimated Dk in Hilafilcon B suggest a greater extent of vitamin C uptake than in Senofilcon A, owing to its greater hydrophilicity and open polymeric structure. Overall, these results demonstrate that while Hilafilcon B provides superior hydration and oxygen permeability, which are beneficial for ocular comfort, the incorporation of vitamin C may slightly decrease these properties, as reflected in the observed water content trends.

To summarize, Table 6 presents a comparative overview of the key properties of Senofilcon A and Hilafilcon B, including vitamin C release behavior, equilibrium water content, and oxygen permeability.

**Table 6:** Summary of Senofilcon A and Hilafilcon B comparison.

Characteristic	Senofilcon A	Hilafilcon B
Vitamin C loading capacity	9.3 µg/mL	12 µg/mL
Best fit release profile	Korsmeyer-Peppas and Higuchi	Korsmeyer-Peppas and Higuchi
Vitamin C release mechanism	Diffusion and polymer matrix swelling-relaxation (anomalous transport)	Diffusion and polymer matrix swelling-relaxation (anomalous transport)
Water content	∼ 27%–32%	∼ 55%–57%
Oxygen permeability, Dk	4.8–5.9 barrer	14.6–16.3 barrer

## 4 Conclusion

This study developed vitamin C-loaded contact lenses using a simple soaking technique and evaluated their incorporation efficiency, release profile, and stability. Two types of commercial lenses, Senofilcon A and Hilafilcon B, were investigated. The results revealed that Hilafilcon B exhibited higher vitamin C loading and release capacity than Senofilcon A, which is attributed to its greater hydrophilicity and water content. The release kinetics of both lenses followed the Korsmeyer-Peppas model, indicating a non-Fickian or anomalous transport mechanism governed by diffusion and polymer relaxation. Vitamin C stability was influenced by storage temperature and UV exposure, with refrigeration providing optimal preservation. The incorporation of vitamin C slightly reduced the water uptake and oxygen permeability of both lenses, suggesting molecular interaction within the hydrogel matrix.

Future investigations should aim to establish the clinical relevance and safety of this system. Sterility testing and *in vitro* cytotoxicity studies on corneal epithelial or fibroblast cells, followed by *in vivo* evaluation, are recommended to verify ocular compatibility and therapeutic potential. Optimization of loading parameters such as vitamin C concentration, pH, soaking duration, and storage conditions may improve drug retention and release longevity. Long-term stability analysis and exploration of alternative hydrogel materials could further enhance performance. These extensions will support the translation of vitamin C-loaded contact lenses into sterile, sustained-release therapeutic platforms for the management of oxidative stress-related ocular disorders.

## Data Availability

The original contributions presented in the study are included in the article/Supplementary Material, further inquiries can be directed to the corresponding author.
